# Hospital and Institutional News

**Published:** 1920-10-23

**Authors:** 


### HOSPITAL AND INSTITUTIONAL NEWS.

### "OUR DAY."

PERHAPS the most pathetic incident of Our Day was the march through Westminster of a thousand wounded soldiers—the men who know the value of the Red Cross better than anyone. Most of these men went by either on crutches or in self-propelling wheelchairs, the useful vehicles which take them long distances—to football matches and elsewhere. The Duke of York devoted most of Thursday to the cause of the Red Cross. He spent an hour at the special matinée at the Pavilion Theatre, then visited the open-air market in the courtyard of Devonshire House (see illustration on this page), from thence to the Central Hall, Westminster, where he was welcomed by nearly 1,200 sick and wounded men who had assembled from the hospitals at the invitation of the British Red Cross Joint Committee to enjoy a concert followed by high tea, the guests being many of those who had joined in the procession described above. The chorus singing of the audience was a great feature, and by request of the Duke several of the items were repeated. The Scots Band had a big share in the success of the entertainment. Throughout Thursday the collectors, many of them nurses, had but small difficulty in disposing of the flags and souvenirs. At Drury Lane a baby camel was very popular as a moving balloon-stall, and another novel feature was a roasted-chestnut barrow. The ingenuity of a crowd of helpers was largely called upon to produce novel features, and it is our sincere hope that all concerned will be rewarded by the announcement of a handsome financial result.

### PRINCE HENRY HELPS ORTHOPÆDIC HOSPITAL.

ALTHOUGH the immediate financial result of the Mansion House meeting was not large, the Royal National Orthopædic Hospital is likely materially to benefit through Prince Henry's advocacy. The Prince made a simple speech, telling his audience that, as he was too young to serve in the great war, he was trying to make up for it by helping such excellent objects as the well-known hospital in Great Portland Street, which had treated 18,000 serving and disabled soldiers, and where the out-patient attendances had multiplied fivefold in eleven years. Seventy-five per cent. of the patients are children, and as treatment in the early stages very often means cure the saving to the public must be considerable. Propaganda at the National Orthopædic is being very vigorously spread, and the management have held several trump cards recently, including a letter from the Prime Minister commending the appeal, and saying how much Mr. Lloyd George is aware of the terrible wastage through the omission of early treatment of deformities.

### THE STRIKE AND THE HOSPITALS.

As we go to press it is too early to give any definite forecast of the ultimate effect upon the hospital world of the miners' most unwise decision. As things stand at the moment, when the immediate prospect of coal shortage might be the chief source of institutional anxiety, we are glad to note that the majority of hospitals are supplied up to a period of several weeks. Also, as long as the transport facilities remain at their present level the questions of food and general supplies are, in the majority of areas, not unduly urgent; but for every day the main strike continues, with more and more workers being thrown out of employment, the possible onset at any time of severer weather and the combined effect in lowering the vitality and health of the people concerned, there will come indirectly a vastly greater strain on the resources, particularly of the provisional hospitals and those serving essentially industrial areas. There is no doubt that many of the recent administrative reorganisations will be tested near to breaking-point. Down south the effects of the strike are barely perceptible at the moment. Large stores of fuel have been everywhere accumulated, and where storage was limited supplies have been freely dumped on the ground in preparation for the worst. But in the Midlands and North country almost immediate distress is anticipated owing to the sudden stoppage of many industries dependent on the coal supply. The newly-introduced system of payment by patients is likely to be severely tested in the districts affected, and will probably break down altogether before the strike has been many days in progress. Never has there been a worse moment for meeting the additional strain which this unhappy strike will speedily lay on all institutions for the sick.

### AMBULANCES AND THE STRIKE.

An interesting feature of the Conference held at Winchester of the ten motor ambulance stations in Hampshire, under the British Red Cross Society's Home Service Scheme, was the statement made by Mr. A. Moray Williams, chief assistant county director, on the services ambulances could render during strikes. The general scheme was to provide the county with an efficient fleet of motor ambulances working in close co-operation, with a head administrative office. Where the food supply was endangered by strikes or the failure of public transport, the central office would place the whole fleet at the local disposal of the public health authority to secure the delivery of food to the county hospitals and infirmaries. In such an emergency he hoped that each station would provide a driver with its car. The present distribution was experimental, and it might be desirable to rearrange them. Their position should be known to every body throughout the county from the police to the post offices, and much time would be saved in cross-country journeys if one station co-operated with another. Motor transport has given a new weapon to the public to protect itself in such emergencies as strikes, and in country districts especially the hospitals, by means of these ambulances, are less at the mercy of strikers than before. The perfection of the Home Service Ambulance Scheme is all the more desirable if the hospitals are to be served without competition between one another at a time of crisis.

### WOMEN MEDICAL STUDENTS.

There seems to be a singularly high proportion of nonsense in recent press accounts of the sex problem in the University College Hospital Medical School. That such things disturb the governing authorities we have no doubt, and there is extraordinarily small need for the press to ferment their difficulties by concocting a lot of "copy" which in many parts is little short of ridiculous. We have often enough, in these columns, urged the fact that a large number of women are fully capable of following an essentially and economically useful career as medical practitioners. On that point there is really little more to be said, as women have established it for themselves and in no uncertain fashion. What really happened at University College Hospital appears to be practically no concern of the public whatever. It was, like the majority of such discussions, merely a question of reformed internal administration. There are a number of instances in collegiate life where, in an institution originally built and adapted for male students, the influx of a large number of women has created no little difficulty in solving accommodation problems. The male students as the original inmates probably have a definite reason to protest that both their space and general comfort are encroached upon in this process. Doubtless the necessary alterations will be made, even at the expense of entire abolition of co-education, but in any case it is emphatically not a case of "down with the women doctors!"

### RETIREMENT OF COLONEL DEANE.

Lieutenant-Colonel Andrew Deane has resigned the post of superintendent of the Royal Victoria Hospital, Belfast, after nineteen years' service. He succeeded Colonel John Glancy in 1901 at the time when the present hospital was being built, and for the first few years of his appointment he worked at the old Royal Hospital in Frederick Street, besides supervising the new building, which was planned in 1897 to commemorate the Diamond Jubilee of Queen Victoria. Under the Mayoralty of Mr. (now Lord) Pirrie £100,000 was raised for the new hospital, and subsequently another large fund for its endowment was successfully started. On July 27, 1903, King Edward opened the present hospital, and named one of the wards after their eldest son, the late Duke of Clarence. Colonel Deane quickly set his mark on the administration of the new institution; he was preoccupied with its welfare and organisation, and staff and patients alike found in him a trusted friend and a devoted administrator. He was an adept at hospital festivities, and the Christmas entertainments were his peculiar delight. The great roll of medical superintendents found in him a fine example. A student at the old Ledwick School of Medicine in Dublin, he qualified in 1869, and in 1881 became M.D. of Durham and an F.R.C.S. of Ireland. A member of the Indian Medical Service, he served in Bengal and other parts of India, and became Inspector-General of the hospitals in the Punjab. His achievements in Belfast include the encouragement of the Working Men's Committee, which is now a very important factor in the hospital's prosperity. He will be hard to replace, and his service has been honourable, both to himself and to the hospital. We trust that his health will recover now that he is relinquishing his duties. The high repute of the hospital to-day is as much his work as anyone's.

### £700,000 FOR EDINBURGH.

The munificent bequest which will shortly become payable to the Edinburgh Royal Infirmary does not come, of course, as a surprise, as it was announced fifteen years ago that Mr. David Ainslie, of Costerton, had left his large fortune to the infirmary. £700,000, however, even in these days of smaller purchasing power, is a goodly sum and should go a long way towards the intention of Mr. Ainslie, which is to provide convalescent homes in connection with the Royal Infirmary.

### THE NEW DEPARTMENT AT BETHLEM.

The opening on October 22 of the new *x*-ray department at the Bethlem Royal Hospital is made more interesting from the monograph written for the occasion by Dr. J. G. Porter-Phillips, the medical superintendent. His reference to the past and to the improvements made of recent years is welcome, since Bethlem does not figure in the public eye and most people know more of its doings in the eighteenth century than of those now carried on within its walls. Last year a new department for nervous diseases was started to enable neurology and psychology to be studied side by side, and the new *x*-ray installation which the hospital's president, Sir C. C. Wakefield, has given will aid the research work always carried on. Bethlem also possesses a complete cinematograph for the amusement and no doubt the instruction of the patients and staff. The former are a class of patients drawn from the educated classes and selected in the earlier stages of disease. They were also furnished in 1896 with a big recreation hall in which a fully equipped stage is provided. In fact this ancient foundation, which was established in 1247, is one of the most up-to-date of any of its kind. Its history has been written by the chaplain, and the old tradition to take sufficient pride in what is done to make a graceful record of it is apparent in Dr. Porter-Phillips' pamphlet on the present occasion.

### PATIENTS' PAYMENTS AT NORWICH.

A grave warning about the possibility of having to close the Norfolk and Norwich Hospital altogether was uttered by the Chairman of the Finance Committee, Mr. Charles Larking, at the quarterly meeting of the governors. The report described measures to be taken to increase the income, and stated that a portion of the annual deficiency should be made up by the patients who receive benefit in the hospital. The Chairman, Mr. Robert Gurney, explained that, instead of payment as in the past being the exception, it should be the rule in future, although patients would be admitted whether they could pay or not, but it was hoped that a large proportion would be able to pay. For the nine months ending September 30 the excess expenditure over income reached £14,500, against a deficiency of £7,000 for the same period last year. In those circumstances the possibility of closing could not be taken as an idle threat. It is obviously necessary that business firms shall recognise their obligation voluntarily unless they prefer extra rates or extra taxation. This does not matter, presumably, to the Norwich Corporation, one of the largest employers of labour in the City, who subscribe only twenty guineas per annum, notwithstanding that the police ambulance brings an average of 1,400 patients to the hospital every year.

### PATIENTS' PAYMENTS AT ST. GEORGE'S HOSPITAL.

In future all in-patients at St. George's Hospital are to contribute 10s. 6d. to £3 3s. per week, according to their means, towards the cost of their maintenance. With the exception of war pensioners and V.D. patients, out-patients are to contribute from 6d. to 2s. per attendance, according to the department. Necessitous cases, both in-patients and out-patients, if found so to be after investigation by the Almoner, will be treated free, as heretofore. The results show that thirty-one per cent. of the patients during five weeks have contributed, and if the same proportion is maintained an additional income of £5,000 is assured.

### THE HONITON HOSPITAL CONTROVERSY.

The lack of hospital accommodation in Honiton and the state of the Honiton and District Rural Nursing Association is exciting local controversy. This is to be welcomed, because the hospital which Honiton had before the war was closed, apparently through lack of funds and enthusiasm, and the Nursing Association seems to be limited to a single district nurse. In these circumstances Dr. Steele Perkins, a local practitioner, has urged the Mayor to call a public meeting, to see if a cottage hospital cannot be revived. He reminds the local Press, which insists on the need for a hospital, that Honiton is growing, and that timber and motor works, in both of which accidents are bound to occur, are being established in the place, and that during the war the military hospital, which extended to fifty beds, was "flooded with money and gifts." On the other side, the Rector of Honiton, as Chairman of the Nursing Association, seeks to explain the closing of the nursing home by want of money and of nurses, and urges that accidents can be taken to Ottery or Exeter. Neither of these arguments seems conclusive, and we hope that the effect of the discussion will be to bring all parties together, so that a hospital may be resuscitated at Honiton with proper support.

### THE SEX OF HOSPITAL PORTERS.

The question of hospital porters was mentioned at the recent meeting of Swansea Hospital Board. Mr. C. Tuckfield declared that the committee had been considering whether to engage male instead of female porters, but could not see its way to revert to the pre-war practice. "Indeed, as a matter of fact," the speaker continued, "women porters do the work much better than it used to be done by men." Is this the general opinion?

### SUNDAY CINEMAS AND HOSPITAL FUNDS.

By the decision of the East Ham Council to refuse permission to the local cinemas to open on Sundays, the Hospital Fund, it is stated, stands to lose some £40 per week—the amount the proprietors were prepared to give to this object had their request been granted.

### SHEFFIELD'S NEW SANATORIUM.

Sheffield Corporation's new sanatorium at Norton House will cost £50,000. This scheme is in lieu of that originally proposed for the Rivelin Valley site, with the difference that the present scheme will be based on utilising certain existing buildings on the site, the new buildings being mainly adapted army huts, as against permanent buildings. The site contains over forty acres and cost £7,500. The plans which have been prepared show accommodation for 136 patients in wards, single and double rooms, in pavilions with accommodation for administrative staff in the existing house, and nurses' home, assistant medical officer's quarters, dining-room, recreation room, and dispensary in timber huts. Existing farm buildings will be adapted as workshops for training suitable patients. It is suggested that the accommodation shall ultimately include forty cottages for the residence of certain patients with their families; but this will be deferred for the present. The scheme has been drafted in consultation with, and in accordance with requirements of, the Ministry of Health, which will contribute at the rate of £180 per bed. The complete scheme is computed to involve a cost of over £90,000.

### A SHEEP A YEAR FROM FARMERS.

The community appeal is proving successful in the Newcastle Royal Victoria Infirmary campaign for funds. Following other sectional organisations, the latest development is the farmers' mart meeting. The mining, shipping, and shipbuilding industries have been successfully worked; agricultural interests in the counties of Northumberland and Durham are now being appealed to. At Rothbury, a market town in the heart of Northumberland, a meeting was recently arranged by the auctioneer, Mr. R. Donkin, at which Lord Armstrong, chairman of the infirmary, presided, and Mr. A. Fletcher, financial secretary, presented the claims of the hospital. Mr. Donkin initiated a scheme in which the farmers of Coquetdale agreed to co-operate to form a fund to be built up on the basis of "a sheep a year" for infirmary funds. The names of fifty farmers were enrolled as contributors at the initial meeting, which was held in the interval of a big sale of sheep. A representative local committee was formed to carry out the proposal. It is intended to provide for a "Coquetdale Farmers' Bed" in the infirmary. A neighbouring mart held at Scots Gap has contributed £173 in response to the appeal, and a Farmers' Manure Association recently sent £35 out of a balance of funds available for charitable purposes. The butchering trade also is likely to prove a productive unit of contribution.

### NEW COTTAGE HOSPITAL AT DOLGELLY.

Mrs. Lloyd George has opened the first Merioneth Red Cross Cottage Hospital at Dolgelly, toward the cost of which the sum of £1,000 has been contributed by the Red Cross Society. The chairman of the hospital's committee is Colonel Enthoven, who stated that Mrs. Lloyd George had narrowly escaped being involved in a motor-car accident on her way to the ceremony.

### SOUTH LONDON HOSPITAL AND EMPLOYERS.

Gradually here and there efforts are being made to enlist the regular support of employers in London. The latest instance is seen in connection with the South London Hospital for Women, where the committee is approaching employers for regular help in consideration of the treatment given to their workpeople and the latter's dependants in the hospital. Even those patients who pay something can rarely do more than give as much as one fourth of the sum which their treatment costs to the institution. London employers are too often indifferent to hospitals, but neither they nor their employees will be successfully organised unless both are approached at the same time. It would be well, therefore, if a beginning were made with those firms which most frequently send patients; and if the employees agreed to hold a weekly collection we think that the heads of the firms would not hold back. In respect of the present appeal, any employers who refuse should be asked to reconsider their decision until their employees have been allowed an opportunity to decide whether to start a weekly collection among themselves.

### BIG LEGACY FOR A COTTAGE HOSPITAL.

The late Mr. Otto Baerlein, once Serbian Consul in Manchester, left the residue of his estate, £20,000, to the Alderley Edge Urban District Council, with power to determine how it should be spent. The Council decided in favour of a cottage hospital, and a site has been chosen on the de Trafford estates for the new Alderley Edge Cottage Hospital.

### THIS WEEK'S DRUG MARKET.

As might be expected, business in the drug market is almost at a standstill, and until the air is cleared no improvement can be anticipated. Buying is restricted to immediate requirements, but there has been no general fall in prices. The most that can be said is that the downward tendency has been somewhat accentuated. Japanese refined camphor is again cheaper, and English refiners have reduced their quotations. Cocaine is offered at lower prices. The firmer tone in bromides that was noticeable a fortnight ago has not been maintained, and there are offers at comparatively low rates. Among synthetic drugs there is a decidedly easier tendency in salicylic acid, sodium salicylate, aspirin, benzoic acid, sodium benzoate and barbitone. The price of methylated spirit has been reduced. Ergot of rye is offered at rather lower rates. Methylated ethers have been slightly reduced in price.

